# The Impact of Shape and Decoration on User Experience and Visual Attention in Anthropomorphic Robot Design

**DOI:** 10.3390/jemr18020005

**Published:** 2025-03-03

**Authors:** Tao Song

**Affiliations:** 1 School of Creative Arts and Design, Zhejiang Polytechnic University of Mechanical and Electrical Engineering, Hangzhou 310053, China; u24092110338@cityu.edu.mo; 2 Faculty of Innovation and Design, City University of Macau, Macau 999078, China

**Keywords:** eye movement, region of interest, attention, art perception, user experience, anthropomorphic robot, shape, decoration

## Abstract

This study aims to explore the effects of Shape and Decoration on user experience and visual attention in anthropomorphic robot design. Eighty undergraduate students were divided into four groups, each viewing one of four stimuli: (a) Non-hat and Non-pattern, (b) Hat and Non-pattern, (c) Non-hat and Pattern, and (d) Hat and Pattern. Eye-tracking data and subjective user experience ratings were collected. The results indicate that both Shape and Decoration have significant effects on user experience and visual attention. The Hat significantly outperformed Non-hat in the dimensions of Attractiveness and Stimulation, while the Pattern showed significant advantages in Stimulation and Novelty. Additionally, Shape and Decoration exhibited a significant interaction effect in the dimensions of Novelty and time to first fixation, suggesting that their combination provides complementary benefits in enhancing perceived novelty and initial visual appeal. Hat and Pattern attracted users’ attention earlier and prolonged fixation time, as seen in time to first fixation, first-pass total fixation duration, and second-pass total fixation duration. For time to first fixation, there was an interaction effect between Shape and Decoration. This study offers strong theoretical support for the design of anthropomorphic robots, highlighting the critical role of Shape and Decoration in user experience.

## 1. Introduction

With the rapid advancement of artificial intelligence and robotics, anthropomorphic robots are increasingly being used in human–robot interaction, service industries, and entertainment. These robots not only perform functional tasks but also engage in emotional interactions with humans, prompting designers to incorporate more cultural and emotional elements into their appearance [1]. Recently, there has been a growing interest in exploring the role of design in modern robotics, particularly the impact of aesthetic-related visual elements on enhancing user experience and emotional resonance [2,3].

In traditional Chinese culture, shape and decoration not only embody aesthetic value but also carry rich historical and cultural connotations [4]. Integrating these traditional elements into the design of anthropomorphic robots can enhance the cultural affinity of robot products and influence users’ cognitive and emotional responses through visual stimulation. However, existing studies mainly focus on the functional design and optimization of interaction technology in anthropomorphic robots [5,6], while research on the impact of traditional shape and decoration on user behavior and experience remains relatively limited. Therefore, this study aims to explore how traditional elements, especially shape and decoration, affect users’ eye movement behavior and user experience to fill this research gap.

### 1.1. Design of Anthropomorphic Robots

Anthropomorphic design refers to the incorporation of human-like appearances, behaviors, or features into non-human objects to enhance interaction with humans [7]. In robot design, anthropomorphism is not merely about making robots look like humans, but also about making them feel more natural and personable in social interactions [2]. By increasing users’ sense of familiarity, trust, and emotional connection, anthropomorphic design significantly influences user interaction experiences [6]. With the growing application of anthropomorphic robots in education, healthcare, and service sectors, researchers are increasingly focused on the impact of robots’ appearance on users’ psychology and behavior [8]. Some studies have shown that the visual attributes of anthropomorphic designs can directly influence users’ cognitive responses and emotional engagement. For instance, features such as facial expressions, gestures, and movements in anthropomorphic robots can facilitate emotional bonding between humans and robots [9,10]. These visual characteristics not only involve biological anthropomorphism but may also include cultural aspects [11]. Therefore, incorporating traditional cultural elements such as shape and decoration into anthropomorphic robot design can enrich their visual layers and enhance cultural affinity.

Shape is a crucial visual element in anthropomorphic robot design. It not only determines the overall appearance of the robot but also influences users’ first impressions and emotional responses. Studies have found that shape design can impact users’ perception and improve the robot’s acceptability and likability [2,6]. Traditional cultural shape elements, such as the streamlined structures and symmetry found in Chinese architecture and attire, can provide novel inspiration for anthropomorphic robot design [12]. These shape elements often carry strong symbolic meanings that evoke users’ cultural memories, thereby strengthening the emotional connection between humans and robots. Traditional elements can endow products with a unique cultural identity, making them more readily accepted and appreciated by specific cultural groups [13].

Decoration is another significant visual element commonly used in product design to enhance visual appeal and cultural expression. Chinese traditional patterns, in particular, convey cultural and historical backgrounds [4]. In robot design, decorative pattern elements can enrich the visual experience of products and enhance users’ emotional connection with the product by evoking cultural memories [14]. Traditional patterns in Chinese culture hold unique symbolic meanings; for example, patterns like dragons and clouds symbolize auspiciousness and power, which can evoke positive emotional responses from users [15]. There is a close relationship between patterns and users’ visual attention. One study found that Chinese traditional patterns enhanced the style and aesthetic performance of chair designs, attracting more visual attention, especially when applied to wooden chairs [16]. Therefore, appropriately incorporating traditional patterns into anthropomorphic robots can influence users’ eye movement behavior and emotional responses through visual stimulation, providing an effective means to enhance user experience.

### 1.2. User Experience

User Experience (UX) refers to the overall feelings and responses people have when using a product or system, encompassing emotional, cognitive, sensory, and behavioral experiences across multiple dimensions [17]. UX theory not only focuses on the functionality of a product but also includes users’ emotional responses and satisfaction during interaction. In anthropomorphic robot design, UX is closely linked to the product’s appearance and interaction experience. Users’ pleasurable experiences often stem from the aesthetic design and emotional value of the product, rather than solely from its functional performance [18,19]. By endowing robots with human-like features such as facial expressions, voice interactions, and anthropomorphic shapes, users can interact with these robots more comfortably, thus enhancing their emotional experience [1]. Particularly, integrating traditional cultural elements into design can evoke users’ sense of cultural identity, strengthening emotional connection and user satisfaction [20]. Traditional patterns and shapes in design not only enhance the robot’s visual appeal but also evoke cultural memories in users, fostering emotional resonance [21]. Such designs help to reduce the perceived distance between users and technological products, making it easier for users to accept robots as companions in their daily lives [22].

Traditional shapes and patterns, as essential design elements, significantly impact users’ visual perception as well as their emotional experience and behavior. Research has found that traditional shapes can endow robots with unique characteristics, giving them stronger cultural recognition, which in turn attracts users’ attention and enhances their willingness to engage [23]. The complexity and symbolism of patterns also affect users’ visual processing, further enriching their experience through visual appeal and the transmission of emotional symbols [16]. Additionally, symbolic patterns play a vital role in enhancing users’ emotional identification. Certain culturally significant patterns, such as traditional Chinese dragon and phoenix motifs, not only attract visually but also convey positive meanings of auspiciousness and harmony, thereby reinforcing users’ psychological security and emotional identification [24]. This makes the robot not just a cold technological product but an entity capable of embodying culture and emotion, which in turn improves user acceptance and satisfaction [25].

### 1.3. Visual Salience Theory and Emotional Design Theory

Visual Salience Theory focuses on the process by which certain elements in a visual scene automatically attract user attention due to their distinctiveness from the surrounding environment. Salient features such as color, size, contrast, and complexity guide users to prioritize specific areas through a “bottom-up” cognitive mechanism, which is particularly crucial in design scenarios that require rapid attention capture [26]. This theory has been widely applied in the field of design, especially in optimizing visual appeal and enhancing user experience. In design practice, salience theory helps designers identify which visual elements can quickly draw users’ attention [27]. For instance, in product design, striking shapes, high-contrast decorations, and intricate patterns can serve as salient design features to direct users’ visual focus. This prioritized attention allocation influences users’ first impressions and subsequent behaviors, thereby enhancing the product’s appeal and user experience [28].

Emotional Design Theory, introduced by [29], emphasizes the role of emotions in shaping users’ interactions with products. This theory posits that users experience products on three levels: visceral (appearance), behavioral (functionality), and reflective (cultural and emotional significance). Emotional responses to design elements can influence users’ overall satisfaction and willingness to engage with a product. Emotional Design Theory is widely applied in fields such as industrial design, user interface design, and product development, particularly in the design of high-value-added products. For users, appearance and functionality are crucial factors influencing product satisfaction and user experience, operating at the visceral and behavioral levels. Moreover, designs incorporating cultural symbols can enhance users’ emotional identification with a product through the reflective level [30]. For example, the French luxury brand Louis Vuitton incorporates its iconic Monogram pattern into handbag designs, allowing users to perceive the brand’s unique value while evoking aspirations for a noble culture and refined lifestyle [31]. Similarly, the application of traditional cultural elements in Chinese chair designs demonstrates that by adding traditional Chinese patterns, users are more likely to develop a sense of cultural belonging and emotional resonance with the product [16].

Visual salience emphasizes the bottom-up mechanism of attention allocation. In Emotional Design Theory, the visceral level leverages these visual features to evoke immediate sensory pleasure in users. This distribution of visual attention can be objectively captured using eye-tracking technology. Consequently, some researchers have attempted to explore the connection between visual attention and design emotions within the design field using eye-tracking methods [32,33]. However, as emphasized in Visual Salience Theory, visual salience cannot be solely described by the physical properties of visual stimuli; the observer’s prior experiences also play a role [26]. Emotional Design Theory highlights the reflective level’s influence on user emotions and attention, underscoring the importance of culture [29]. Culturally derived stimuli may also impact visual appeal. Nevertheless, the role of culture in visual appeal remains underexplored, especially in studies that utilize eye-tracking technology to collect objective visual data and integrate Visual Salience Theory with Emotional Design Theory. Such research addressing this intersection is still relatively scarce.

### 1.4. Eye-Tracking Research

Eye-tracking technology involves recording and analyzing eye movement trajectories to study visual attention distribution and cognitive processes when individuals observe objects or scenes [34,35]. This technology is widely used in fields such as psychology, cognitive science, market research, and human–computer interaction [36,37,38,39,40]. By measuring data such as users’ fixation points, fixation duration, pupil size, and saccades, researchers can understand users’ responses to specific visual stimuli, revealing their attention distribution, emotional responses, and information-processing patterns [41,42]. In anthropomorphic robot design, eye tracking can help designers evaluate the impact of visual elements, such as the robot’s appearance and pattern designs, on users’ visual attention and emotional experiences.

Visual attention refers to the process by which individuals selectively focus on certain information while ignoring others in a complex visual scene [43,44]. Visual attention is generally a physiological manifestation of user interest or cognitive load. When observing design works, interest often reflects aesthetic experience, whereas cognitive load is associated with the complexity of design elements. Users’ subjective interest can be inferred from eye movement metrics. When design elements are sufficiently interesting and salient, they are detected more quickly by users. In eye-tracking experiments, when the complexity of stimuli is controlled to ensure participants are not distracted, time to first fixation can serve as a reliable indicator of how quickly a design element captures users’ attention [45,46]. Many researchers use total fixation duration as a metric to interpret the degree of user interest in specific areas. However, this approach has limitations: An increase in visual attention does not always reflect a positive experience related to user interest. Instead, it may be influenced by the complexity of the information and the cognitive process of decoding it. Researchers have employed two methods to address this limitation. The first approach combines objective eye-tracking data with subjective data, which helps address the inability of fixation duration alone to explain its sources of variation. For example, in a study on Chinese chair designs, the researchers collected both eye-tracking data and subjective evaluation results [16]. The findings showed that participants’ visual attention was concentrated on areas with traditional patterns, a result consistent with their aesthetic evaluations. The second approach involves improving the analysis of eye-tracking data. Some researchers have proposed dividing total fixation duration into two components: first-pass total fixation duration and second-pass total fixation duration [47,48]. The former refers to the duration from the first entry of the eye into a specific area of interest to its first exit. This metric reflects the user’s allocation of attention during the initial exploration of visual elements within the area. While attention distribution during the first pass is influenced by both interest and the difficulty of decoding information, the former plays a more significant role. A higher value for this metric indicates greater interest [48]. The second-pass total fixation duration, on the other hand, refers to the sum of all fixation durations after the first pass. Compared to the first pass, this metric reflects the richness of the information or the difficulty of decoding it [47]. Analyzing these two metrics together provides a more comprehensive understanding of users’ visual attention distribution.

In anthropomorphic robot design, the study of visual attention is particularly important, as various design elements of the robot’s appearance—such as shape, patterns, and color—can influence users’ first impressions and subsequent interaction behavior. The introduction of traditional elements may further affect users’ visual attention distribution because these elements can trigger cultural memories and emotional responses, thereby altering their visual scanning paths and fixation patterns [16,49]. Shape and pattern, as essential visual design elements, have a direct impact on users’ eye movement behavior. Shape defines the overall contour of the design, while pattern adds visual depth and cultural significance through detailed design. Together, they influence user experience while also capturing visual attention [33,50]. By analyzing eye-tracking data alongside subjective evaluation data, we can gain deeper insights into the appeal of design elements and users’ experiences. This provides valuable support for designers to optimize design decisions and improve user experience.

### 1.5. The Design

Some scholars and designers have begun focusing on the integration of traditional cultural elements with modern technological products. This fusion, especially in anthropomorphic product design, has demonstrated wide-ranging potential for applications [51,52]. Traditional elements not only add aesthetic value to modern technological products but also enhance cultural affinity, which in turn improves users’ emotional experience and satisfaction. Designs featuring traditional cultural elements often evoke users’ sense of cultural identity and pride, thereby increasing their acceptance of the product [53].

**Figure 1:**
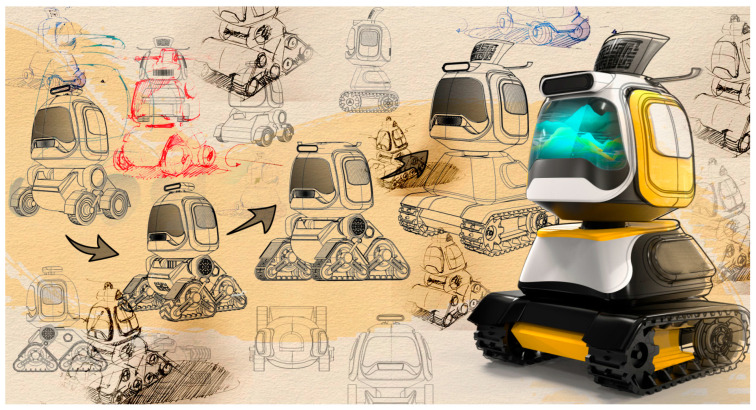
Partial design sketches of the anthropomorphic robot.

**Figure 2:**
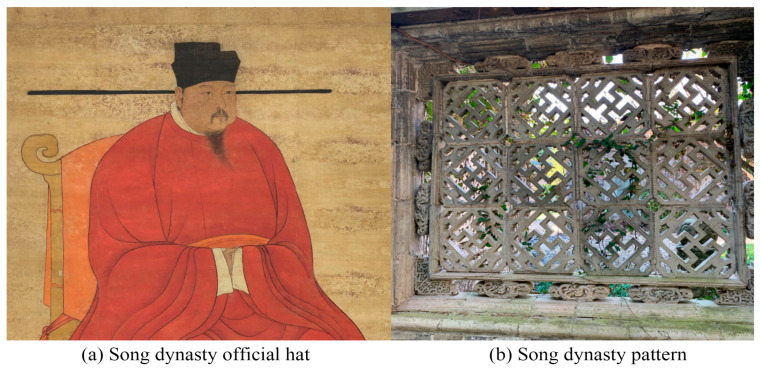
Sources of Chinese elements used in the robot design: (**a**) Song Dynasty official hat and (**b**) Song Dynasty pattern.

**Figure 3:**
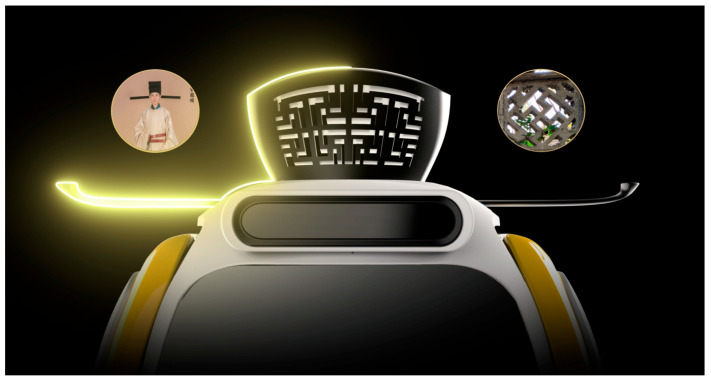
Application of traditional Chinese elements on the anthropomorphic robot.

The anthropomorphic robot in this study was designed for a commercial center in China themed around the Song Dynasty (a feudal dynasty in Chinese history). The robot is intended to provide information and emotional experiences to visitors. This robot is equipped with functions such as visual information display, conversation, mobility, and intelligent control. Given the context of use and the characteristics of the users, the design of this anthropomorphic robot needs to meet functional requirements while also reflecting the unique culture of the Song Dynasty. Some of the initial sketch designs are shown in Figure 1, where arrows indicate the evolution of the robot’s form. The design mainly incorporates two traditional Chinese elements: the Song Dynasty official hat and the Song Dynasty pattern (Figure 2). The Song Dynasty official hat is a hat traditionally worn by government officials of the Song Dynasty, known for its simplicity and elegance. The shape of the official hat has been utilized in many products, such as chairs, and is well-recognized among Chinese people. The Song Dynasty pattern is inspired by the ornamental stone carvings found in parks from the Southern Song period, symbolizing good fortune and prosperity. As a visual element, traditional Chinese auspicious patterns are widely used in visual communication design, product design, and environmental design and are very popular in China. The Song Dynasty official hat and the Song Dynasty pattern are incorporated into the head of the anthropomorphic robot (Figure 3).

### 1.6. The Present Study

There is an undeniable correlation between product design and both user experience and visual attention, as confirmed by numerous studies. While these studies may focus on different types of products or adopt various design approaches, they consistently emphasize the importance of design elements. For instance, one study explored the shape and decoration of Chinese Tang dynasty chairs, revealing their relationship with emotional evaluations and their impact on eye movement [54]. When it comes to shape, chair design particularly emphasizes the form of the backrest [55], while graphic designers focus on how the shape of typography affects attention in graphic advertisements [56]. An eye-tracking study on transportation found that professionals tend to focus on functional aspects, whereas non-professionals are more influenced by decorative elements. The impact of decoration on user experience is also evident in interior design [57]. Furthermore, other research has examined how material and color influence users’ visual behavior and aesthetic experiences [58]. In studies involving user experience and eye-tracking behavior, traditional Chinese elements have frequently been highlighted [16,54]. When selecting traditional elements, designers and researchers need to consider the characteristics of the product and its application context. Overall, while these studies arrive at varied conclusions, they collectively validate that specific design elements can enhance user experience and attract faster or greater attention to these features. Moreover, some studies analyze the interaction effects of multiple factors, demonstrating the complexity of how design impacts users.

Existing research provides valuable insights into anthropomorphic robot design, the integration of traditional cultural elements, and the influence on eye movement behavior. However, there remain certain research gaps and limitations. Many robot studies primarily focus on the functional aspects of design and the technological dimensions of human-robot interaction, while research considering design elements such as shape and decoration is rarely conducted in the field of robotics. The impact of traditional cultural elements in the visual design of robots on user behavior and emotional responses—particularly how shape and decoration influence users’ eye movement behavior—has been relatively underexplored. In the fields of eye-tracking and product design, while Visual Salience Theory [26] and Emotional Design Theory [29] can to some extent help researchers explain users’ visual behavior and experience, there is a lack of theoretical advancement. Additionally, insufficient attention has been given to the role of culture in shaping product-related emotions and visual attention, as well as the integration of these two theories within eye-tracking research. This study aims to explore whether the incorporation of traditional Chinese elements into anthropomorphic robots affects user experience and to examine how these elements influence users’ visual attention across different designs. On the one hand, this research contributes to the fields of user experience and visual cognition; on the other hand, it offers new insights into the design of anthropomorphic robots.

This study addresses the following two research questions (RQs):

**RQ1**: *Do shape and decoration have significant independent or interactive effects on user experience? We hypothesize that both shape and decoration enhance user experience, but there is no interaction effect between them.*

**RQ2**: *Do shape and decoration have significant independent or interactive effects on visual attention? We hypothesize that both shape and decoration reduce the time to first fixation, increase the first-pass total fixation duration, and increase the second-pass total fixation duration. Shape and decoration do not have an interaction effect on the eye-tracking metrics.*

## 2. Materials and Methods

This is a quantitative study with shape and decoration as the independent variables, utilizing a 2 (Hat, Non-hat) × 2 (Pattern, Non-pattern) between-subjects design. As mentioned earlier, shape and decoration are important design elements in products [2,59]. Considering the application scenario of the anthropomorphic robot in this study—used in a Song Dynasty-themed commercial center—two iconic Song Dynasty elements (the Song Dynasty hat and the Song Dynasty pattern) were chosen as key design features and served as the basis for defining the two levels of the independent variables. Incorporating traditional elements into the design not only influences users’ visual attention [16] but also enhances the user experience [53]. When users are familiar with the traditional elements used in a product, they are more likely to resonate with it, which further impacts their visual behavior and subjective perceptions [4,60]. The 2 × 2 design adopted in this study is based on users’ cognitive characteristics and the context of Chinese culture. The dependent variables are user experience, time to first fixation, first-pass total fixation duration, and second-pass total fixation duration. This design enables us to address whether shape and decoration independently or interactively affect visual attention and subjective experience.

### 2.1. Participants

A total of 80 undergraduate students participated in this study. None of them were design majors. Their average age was 20.16 years (SD = 1.65), with 47 female participants. All participants had normal or corrected-to-normal vision. Prior to participation, they provided informed consent. After completing the experiment, they received a gift valued at USD 5 as compensation.

### 2.2. Stimuli

**Figure 4:**
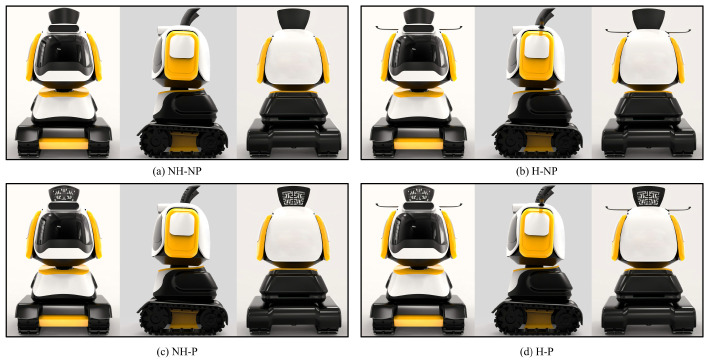
The four designs used as stimuli: (**a**) Non-hat and Non-pattern, (**b**) Hat and Non-pattern, (**c**) Non-hat and Pattern, and (**d**) Hat and Pattern.

To explore the effects of traditional shapes and patterns, four anthropomorphic robot designs were used as stimuli in this experiment (Figure 4). The four designs, created and illustrated by the researchers, included (a) Non-hat and Non-pattern, (b) Hat and Non-pattern, (c) Non-hat and Pattern, and (d) Hat and Pattern. Apart from the hat and pattern elements, all other parts of the four anthropomorphic robots were identical. Each design was presented with a front view, side view, and back view.

**Figure 5:**
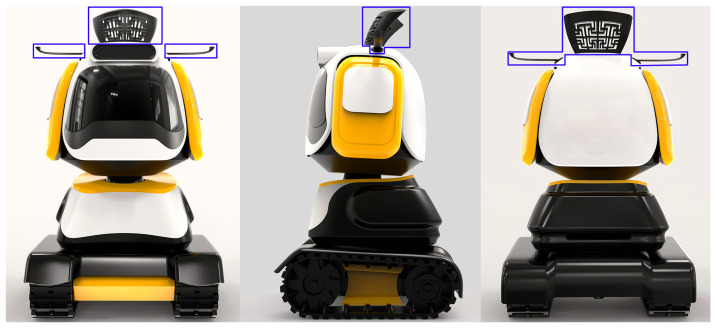
AOI in the stimuli, using the H-P design as an example (blue box indicates the AOI).

An Area of Interest (AOI) was defined on the upper part of each anthropomorphic robot, where the design variations were located. The location and size of the AOI were consistent across all four designs. Figure 5 illustrates the AOI in the H-P design.

### 2.3. Equipment

The User Experience Questionnaire (UEQ) was used to assess participants’ perceptions of the anthropomorphic robot designs [61]. The questionnaire consists of 26 pairs of semantically opposite adjectives, each describing an attribute of the product. Each pair is rated on a 7-point scale, represented by circles between the opposing terms. For example, the pair annoying and enjoyable is rated such that 1 represents very annoying and 7 represents very enjoyable. The 26 items construct six dimensions: Attractiveness, Perspicuity, Efficiency, Dependability, Stimulation, and Novelty. The Chinese version of the User Experience Questionnaire was used, and a pilot study yielded a Cronbach’s alpha of 0.86. The UEQ is widely used to measure the user experience of interactive products [62]. A chatbot was developed and the researchers chose the UEQ for its evaluation [63]. The study selected the UEQ because it is a questionnaire that users can answer quickly while considering both hedonic and pragmatic aspects, effectively reflecting users’ acceptance of robotic products. Specifically, Perspicuity, Efficiency, and Dependability correspond to the robot’s pragmatic quality, while Attractiveness, Stimulation, and Novelty represent its hedonic quality. This dual perspective enables researchers to discuss user experience with the product from both pragmatic and hedonic dimensions. Eye movement data were collected using a Tobii Pro Fusion eye tracker (Tobii, Stockholm, Sweden). A 21-inch monitor was used to display the stimuli.

### 2.4. Procedure

**Figure 6:**
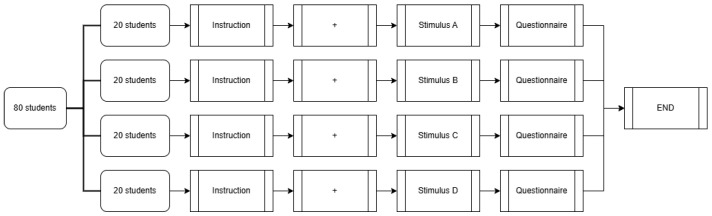
Experimental procedure flowchart.

The 80 students were randomly assigned to one of four groups, with the experimental procedure being identical except for the stimulus viewed (Figure 6). After the researcher provided participants with basic information about the study and explained the key points to consider during the experiment, participants entered a closed and soundproof room. On the desk was a monitor for displaying the stimuli, a keyboard, and a printed questionnaire. The experiment instructions were displayed on the screen. After fully understanding the instructions, participants pressed the space bar on the keyboard, which presented a “+” symbol at the center of the screen. According to the researcher’s guidance and the instructions, participants focused their eyes on the “+” symbol. After 1000 ms, the stimulus was automatically displayed on the screen. The stimuli shown to the participants were either (a) Non-hat and Non-pattern, (b) Hat and Non-pattern, (c) Non-hat and Pattern, or (d) Hat and Pattern. Eye movement during the viewing of the stimulus was recorded by the eye tracker. After viewing the stimulus, participants completed the questionnaire.

### 2.5. Data Analysis

The eye movement metrics used in this study included time to first fixation, first-pass total fixation duration, and second-pass total fixation duration (Table 1). Eye movement data were processed and exported using Tobii Pro Lab (Tobii, Stockholm, Sweden). The questionnaire data were manually transcribed by the researchers into electronic spreadsheets. Statistical analyses were performed using SPSS 26.0. Descriptive statistics were calculated for the total user experience score, the scores for each of the six dimensions, time to first fixation, first-pass total fixation duration, and second-pass total fixation duration. Shape and decoration were treated as independent variables, and main effects and interaction effects were analyzed. Simple effects were further analyzed in the presence of significant interaction effects. A significance level of *p* < 0.05 was adopted for all analyses.

## 3. Results

### 3.1. User Experience

**Figure 7:**
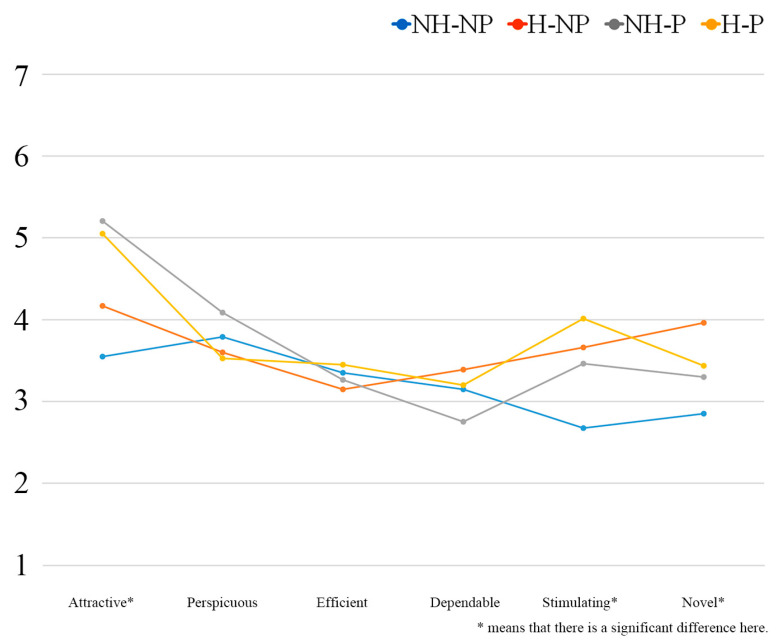
Six dimensions of user experience for the four designs.

The descriptive results for user experience are presented in Table 2. The main effect of Shape was significant, with Hat (M = 3.7 ± 0.42) being significantly greater than Non-hat (M = 3.4 ± 0.46), F (1, 76) = 9.091, *p* = 0.003, η^2^ = 0.107. The main effect of Decoration was also significant, with Pattern (M = 3.7 ± 0.35) significantly greater than Non-pattern (M = 3.5 ± 0.52), F (1, 76) = 8.143, *p* = 0.006, η^2^ = 0.097. The interaction effect between Shape and Decoration was not significant, *p* > 0.05. The user experience results for the six dimensions are shown in Figure 7.

The descriptive results for the six dimensions of user experience across the four designs are shown in Table 3. We further analyzed the main effects and interaction effects for each dimension.

In the dimension of Attractiveness, the main effect of Shape was significant, with Hat (M = 5.1 ± 1.20) being significantly greater than Non-hat (M = 3.9 ± 1.07), F (1, 76) = 25.571, *p* < 0.001, η^2^ = 0.252. There were no significant main effects or interaction effects of Decoration (*p* > 0.05).

In the dimension of Stimulation, the main effect of Shape was significant, with Hat (M = 3.8 ± 1.32) being significantly greater than Non-hat (M = 3.2 ± 1.05), F (1, 76) = 4.926, *p* = 0.029, η^2^ = 0.061. The main effect of Decoration was also significant, with Pattern (M = 3.9 ± 1.16) being significantly greater than Non-pattern (M = 3.1 ± 1.16), F (1, 76) = 8.934, *p* = 0.004, η^2^ = 0.105. However, the interaction effect between Shape and Decoration was not significant (*p* > 0.05).

**Figure 8:**
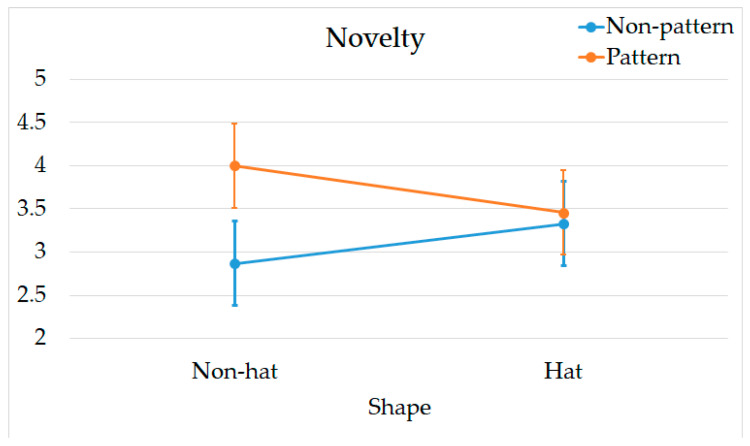
Interaction effects in novelty.

In the dimension of Novelty, the main effect of Shape was not significant (*p* > 0.05), while the main effect of Decoration was significant, with Pattern (M = 3.7 ± 1.30) being significantly greater than Non-pattern (M = 3.1 ± 0.90), F (1, 76) = 6.318, *p* = 0.014, η^2^ = 0.077. A significant interaction effect between Shape and Decoration was observed, F (1, 76) = 4.027, *p* = 0.048, η^2^ = 0.050 (Figure 8). Table 4 shows the simple effects in Novelty.

In the other three dimensions (Perspicuity, Efficiency, and Dependability), no significant main effects or interaction effects were found (*p* > 0.05).

### 3.2. Eye Movement

**Figure 9:**
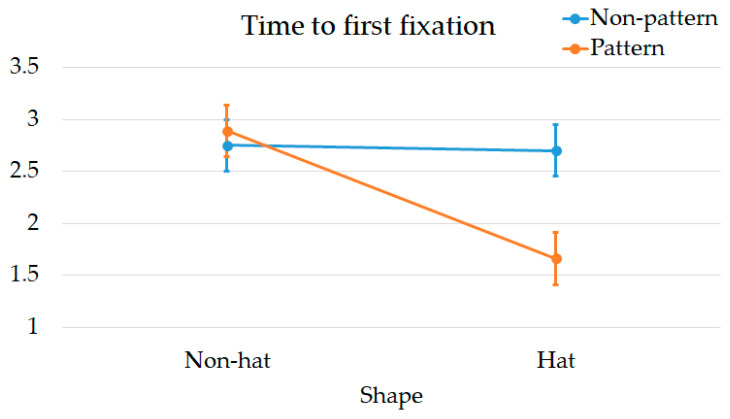
Interaction effects in time to first fixation.

The descriptive statistics for time to first fixation are presented in Table 5. The main effect of Shape was significant, with Hat (M = 2.2 ± 0.69) being significantly less than Non-hat (M = 2.8 ± 0.62), F (1, 76) = 26.797, *p* < 0.001, η^2^ = 0.956. The main effect of Decoration was also significant, with Pattern (M = 2.3 ± 0.85) significantly less than Non-pattern (M = 2.7 ± 0.50), F (1, 76) = 13.204, *p* = 0.001, η^2^ = 0.148. The interaction effect between Shape and Decoration was significant, F (1, 76) = 22.758, *p* < 0.001, η^2^ = 0.230 (Figure 9).

Table 6 shows the simple effects within time to first fixation. Within the Pattern design, Hat (M = 1.7 ± 0.47) was significantly less than Non-hat (M = 2.9 ± 0.69), F (1, 76) = 49.473, *p* < 0.001, η^2^ = 0.394. Within the Hat design, Pattern (M = 1.7 ± 0.47) was significantly less than Non-pattern (M = 2.7 ± 0.46), F (1, 76) = 35.316, *p* < 0.001, η^2^ = 0.317.

The descriptive statistics for first-pass total fixation duration are shown in Table 7. The main effect of Shape was significant, with Hat (M = 2.1 ± 0.79) being significantly greater than Non-hat (M = 1.6 ± 0.71), F (1, 76) = 13.414, *p* < 0.001, η^2^ = 0.906. The main effect of Decoration was also significant, with Pattern (M = 2.3 ± 0.67) being significantly greater than Non-pattern (M = 1.4 ± 0.67), F (1, 76) = 38.295, *p* < 0.001, η^2^ = 0.335. The interaction effect between Shape and Decoration was not significant, *p* > 0.05.

The descriptive statistics for second-pass total fixation duration are shown in Table 8. The main effect of Shape was significant, with Hat (M = 6.4 ± 1.77) being significantly greater than Non-hat (M = 4.7 ± 1.66), F (1, 76) = 28.369, *p* < 0.001, η^2^ = 0.272. The main effect of Decoration was also significant, with Pattern (M = 6.5 ± 1.78) being significantly greater than Non-pattern (M = 4.6 ± 1.54), F (1, 76) = 35.023, *p* < 0.001, η^2^ = 0.315. The interaction effect between Shape and Decoration was not significant, *p* > 0.05.

## 4. Discussion

This study examined the effects of Shape and Decoration on user experience and visual attention through user experience questionnaires and eye-tracking data. The results indicated that both Shape and Decoration had significant main effects on user experience and visual attention. The Hat design was significantly higher than Non-hat in terms of overall user experience ratings and visual appeal, while Pattern was also significantly preferred over Non-pattern. Additionally, although the interaction effects between Shape and Decoration were not significant in overall user experience and most eye-tracking metrics, there were significant interactions in the Novelty dimension and time to first fixation. This suggests that certain combinations of Shape and Decoration may have unique impacts on specific aspects of the user experience. The following sections provide a detailed discussion of these results.

### 4.1. Impact of Shape and Decoration on User Experience

The effects of Shape and Decoration on user experience show both similarities and differences. Both Hat and Pattern have positive impacts on user experience, a finding consistent with previous studies. A product’s shape is often a critical factor in forming users’ first impressions [64]. Decorative elements, especially those with cultural or aesthetic symbolism, can also enhance a product’s visual appeal and emotional value. Hat design may have enhanced users’ aesthetic appreciation and emotional resonance with the anthropomorphic robot, while the complexity and symbolism of the Pattern design significantly heightened users’ emotional arousal, encouraging more active interaction with the product. According to the theory of emotional design, such meaningful designs can evoke users’ emotional responses [29].

Additionally, we observed that among the six dimensions of user experience, only attractiveness, stimulation, and novelty demonstrated significance. These three dimensions correspond to the hedonic quality of user experience, while the other three dimensions, which represent pragmatic quality, showed no significance. This suggests that decorative elements serve not only an ornamental role but also provide additional visual stimuli that can evoke users’ desire to explore and foster emotional resonance [65]. The designs of Hat and Pattern primarily influence users’ experiences on an emotional level [29].

Both Shape and Decoration significantly influenced the results for stimulation. Stimulation is typically associated with users’ interest, curiosity, and desire to explore [66]. A study suggests that complex and meaningfully designed shapes can elicit emotional responses, thereby enhancing user engagement and experience [67]. The Hat design and Pattern, with their traditional Chinese styling, may evoke users’ curiosity, which can evoke users’ desire to explore and foster emotional resonance [65]. This emotional response is consistent with theories suggesting that complex designs enhance the emotional experience by providing greater stimulation, leading to stronger emotional and cognitive engagement [14].

In attractiveness and novelty, the two factors produced different results: Shape influenced attractiveness, while Decoration influenced novelty. These results suggest that although both Shape and Decoration affect the hedonic aspects of user experience, they operate through different dimensions [68]. Visual aesthetics are a crucial aspect of product design, and the Hat design may enhance overall appeal by adding symmetry and contour to the product’s form [69]. Compared to the Non-hat design, the structural design of the Hat may generate a stronger sense of visual attraction and sensory pleasure for users. In this regard, the effect of Pattern was not significant, possibly because the contour modifications brought by the Hat design more readily contribute to users’ perception of the robot’s attractiveness. Additionally, the significance observed in the novelty dimension indicates that the Pattern design provides a novel sensory experience for users. Novelty is a key factor in user experience, particularly in product design, where users are often more attracted to products with unique and innovative features [29]. By incorporating traditional patterns or decorative elements, the Pattern design not only adds visual diversity to the product but also enhances perceived novelty by evoking cultural identity and recognition [15]. This sense of novelty helps create more lasting memories, thereby increasing product acceptance and satisfaction [70].

Moreover, in the *novelty* dimension, Shape and Decoration demonstrated an interaction effect. This interaction was evident in the Non-hat condition, where the Pattern design significantly outperformed the Non-pattern design. This finding indicates that the effectiveness of decorative elements may be moderated by the shape design. In the Hat design, the Hat itself is a prominent visual feature that sufficiently captures attention and enhances perceived novelty, thus diminishing the relative impact of decoration [29]. In the absence of a Hat, the Pattern decoration becomes more salient, thereby increasing the perceived novelty [15].

### 4.2. Impact of Shape and Decoration on Visual Attention

Participants’ eye movements were influenced by both Shape and Decoration, as evidenced by their effects on time to first fixation, first-pass total fixation duration, and second-pass total fixation duration. Overall, both Hat and Pattern positively impacted the ability to attract users’ attention. They not only enabled participants to notice specific design elements more quickly but also increased the time spent focusing on these elements, whether during the initial or subsequent viewing rounds. Under uniform attention interference, larger, brighter, and faster-moving objects are more likely to be perceived, indicating that “bottom-up” attention factors automatically draw focus to prominent visual elements [71,72]. Although previous studies have not specifically examined the effects of Hat shapes and Pattern decorations in anthropomorphic robots, prior research on the mechanisms of shape and pattern influencing visual attention provides valuable references for this study. Complex decorative elements quickly capture users’ attention and sustain their visual interest [16]. Similar effects are observed with Shape [64]. The Hat design may enhance the structural characteristics of the robot’s appearance, making it easier for users to perceive visual information, thus reducing search time. It increases the density and complexity of visual information, requiring users to engage more cognitive resources during initial and subsequent viewings to process the information [73]. In the Pattern design, users may need more time to interpret and understand the symbolic meaning of the patterns, which accounts for the prolonged fixation time. Additionally, due to the cultural and aesthetic characteristics of decoration, users are willing to invest more visual attention in areas featuring patterns.

In time to first fixation, Shape and Decoration also exhibited interaction effects. According to the study results, the ability of Shape or Decoration to shorten participants’ detection time was significant only when one of the design elements was already present. This suggests that in terms of quickly capturing attention, the Hat and Pattern designs must be combined to achieve meaningful results. The combination of Hat and Pattern effectively compensates for the lack of visual appeal caused by monotonous designs [65] However, for fixation duration, there was no interaction effect between Shape and Decoration. Both Hat and Pattern independently enhanced the appeal of the anthropomorphic robot.

### 4.3. General Discussion

Designs incorporating hats (Hat) and traditional patterns (Pattern) performed better in stimulation results and also influenced visual attention: time to first fixation decreased, and fixation duration increased. This aligns with the emphasis of Visual Salience Theory, which highlights how salient features such as color, contrast, and shape automatically capture users’ attention [26]. At the same time, this finding is consistent with the visceral level of Emotional Design Theory, where users focus on the product’s appearance [29]. The user experience results further illustrate the differing roles of shape and decoration in the dimensions of attractiveness and novelty. Hat designs significantly increased attractiveness, while Pattern designs enhanced novelty. Changes in a product’s appearance that influence visual attention should, in theory, benefit the product. However, Visual Salience Theory only explains the process of quickly attracting users’ visual attention and does not address whether the features contributing to attractiveness provide a pleasurable experience for users. For instance, alcohol-related cues in advertisements garner more attention than messages promoting responsible drinking [74], but this outcome is detrimental. Emotional Design Theory complements this limitation. By examining user experience across multiple dimensions, we found that the positive effects of the two different designs stem from different aspects. This finding offers practical guidance for design practice, emphasizing that the integration of shape and decoration can create distinct yet complementary impacts on user experience and visual attention.

In the user experience results, no significant differences were observed for dimensions related to functionality (perspicuity, efficiency, and dependability) across shape and decoration. This outcome is expected since neither design contributed to the functional aspects of the anthropomorphic robot. This indicates that, among the three levels of user experience, there was no difference at the behavioral level (functionality). The Hat and Pattern designs were inspired by traditional Chinese elements, reflecting the application and representation of culture in design [21]. This aligns with the reflective level in Emotional Design Theory. In the user experience results, the incorporation of traditional Chinese culture into the design of the anthropomorphic robot demonstrated its value through the dimensions of attractiveness and novelty. This positive emotional experience in design is further reflected in visual behavior, as designs featuring traditional Chinese elements were discovered more quickly and attracted greater attention.

Visual Salience Theory and Emotional Design Theory complement each other in enhancing product design and user experience. Salient features automatically capture users’ attention, laying a foundation for emotional responses. Users’ focus on the Hat and Pattern areas not only aligns with the predictions of the salience map but also supports the reflective level in Emotional Design Theory. Emotional design enhances the appeal of salient features through cultural symbols and aesthetic value. Culturally enriched designs not only increase users’ visual engagement but also enhance their emotional significance through cultural connotations, transforming these features into both visual focal points and emotional bridges within the user experience.

### 4.4. Implications

The findings of this study have implications for product design and user experience, particularly in exploring the impact of traditional Shape and Decoration on users’ emotional responses and visual attention. These insights provide empirical support for anthropomorphic robot design and suggest several practical recommendations for future product design.

The study demonstrates that traditional Chinese design elements have a significantly positive effect on attracting users’ visual attention and enhancing user experience. This provides strong empirical support for integrating traditional cultural elements into the design of modern anthropomorphic robots. In design practice, designers should focus on the overall shape and the details of decorative elements. By effectively combining shape and decoration, designers can enhance the appeal, stimulation, and novelty of products [29]. For instance, a design with a distinct shape can attract attention early on, while moderately complex decoration can further stimulate user interest and emotional response. By identifying and optimizing specific dimensions of user experience, designers can better meet user needs. For example, products aiming for high visual appeal should prioritize shape design, while products emphasizing emotional experience or cultural symbols can enhance users’ emotional involvement and cultural resonance through complex decorative elements [15]. Shape and decoration also have a complementary role in design. This suggests that when using complex decorative elements, designers should consider the impact of the overall shape of the product. Particularly when seeking to enhance the perceived novelty of anthropomorphic robots or quickly capture users’ attention, an optimal combination of shape and decoration may yield the best results [68].

### 4.5. Limitations and Future Work

While this study provides valuable insights into the effects of Shape and Decoration on user experience and visual attention in anthropomorphic robot design, it has certain limitations that future research should address and improve upon. Firstly, the sample size was relatively small, and the cultural background and age range of the participants were limited, which may restrict the generalizability of the findings. The study utilized a laboratory setting to collect user experience questionnaires and eye-tracking data. Although this controlled environment helps reduce confounding factors, it may not fully reflect user behavior and perceptions in real-life scenarios. This study focused on the impact of Shape and Decoration on user experience and visual attention, but other important design factors, such as color, material, and interaction mode, may also significantly influence users’ perceptions and behavior. For the two independent variables, Shape and Decoration, their two levels inevitably result in changes to the area occupied by the anthropomorphic robot in the stimuli, which may introduce some degree of confounding. Although this change in area can be considered a dimension of Shape or Decoration, it may still affect the interpretation of the results. Finally, this study was quantitative in nature; while significant results were observed, qualitative research is needed to further explain how these effects occur and explore the underlying psychological processes.

We will continue to explore the field of anthropomorphic robot design and its impact on user experience and visual attention. The limitations present in the current research framework will serve as the focal points for breakthroughs in our future work. Achieving higher reliability and validity will be a key consideration, including improving the representativeness of samples, the effectiveness of statistical analysis, and the control of confounding variables during experiments. The selection and control of independent variables in research design will be a primary issue we aim to address. On the one hand, this requires taking into account the practical application scenarios of anthropomorphic robots and the characteristics of their user groups. On the other hand, it involves minimizing the interference of irrelevant factors between different levels of independent variables. In terms of research methodology, we will actively incorporate qualitative research and explore the use of additional tools to analyze and discuss this issue from multiple perspectives.

## 5. Conclusions

This study examined the roles of Shape and Decoration in anthropomorphic robot design, focusing on their effects on user experience and visual attention. By combining user experience questionnaires and eye-tracking technology, relevant data were collected and further analyzed. The results showed that the Hat shape positively influenced attractiveness and stimulation, while the Pattern decoration enhanced user experience in terms of stimulation and novelty. Interaction effects were also observed in the Novelty dimension. Regarding visual attention, it was found that the Hat shape and Pattern decoration could quickly capture initial user attention and sustain engagement through extended fixation durations. In terms of time to first fixation, the interaction between Shape and Decoration highlighted their complementary effects. This study integrates Visual Salience Theory and Emotional Design Theory, offering new perspectives for research on product design and user experience. It emphasizes the connection between the visceral level of user experience and visual stimuli, while validating the impact of cultural elements on the reflective level in design and their manifestation in visual attention. This study offers practical implications for future design practices, emphasizing the crucial roles of shape and decoration in user experience, particularly in enhancing emotional engagement and visual appeal. The findings suggest that product design should integrate the characteristics of shape and decoration to optimize user experience, especially concerning visual attractiveness and emotional resonance. Additionally, this study provides empirical support for further exploring the combination of cultural elements with modern design, revealing the importance of traditional Chinese elements in influencing user experience.

## Figures and Tables

**Table 1:** Description of eye movement metrics.

Eye Movement Metrics	Description
Time to first fixation	Time taken from the start until the first fixation appears within the AOI
First-pass total fixation duration	Duration of fixation during the first entry into the AOI
Second-pass total fixation duration	Duration of all fixations after the first entry, including subsequent entries into the AOI
Eye movement metrics	Description

**Table 2:** Descriptive statistics of user experience for the four designs.

Variable		M	SD	N
Non-hat	Non-pattern	3.2	0.49	20
	Pattern	3.7	0.30	20
	Total	3.4	0.46	40
Hat	Non-pattern	3.7	0.45	20
	Pattern	3.8	0.38	20
	Total	3.7	0.42	40
Total	Non-pattern	3.5	0.52	40
	Pattern	3.7	0.35	40
	Total	3.6	0.46	80

**Table 3:** Descriptive results of the six dimensions of user experience for the four designs.

Variable		Attractiveness		Perspicuity		Efficiency		Dependability		Stimulation		Novelty	
		M	SD	M	SD	M	SD	M	SD	M	SD	M	SD
Non-hat	Non-pattern	3.6	1.02	3.8	1.07	3.4	0.92	3.2	1.13	2.7	0.76	2.9	0.84
	Pattern	4.2	1.06	3.6	0.95	3.2	1.04	3.4	1.06	3.7	1.07	4.0	1.29
	Total	3.9	1.07	3.7	1.00	3.3	0.97	3.3	1.09	3.2	1.05	3.4	1.22
Hat	Non-pattern	5.2	1.22	4.1	1.22	3.3	0.88	2.8	0.93	3.5	1.36	3.3	0.92
	Pattern	5.1	1.21	3.6	0.94	3.5	0.86	3.2	1.10	4.0	1.24	3.5	1.28
	Total	5.1	1.20	3.8	1.11	3.4	0.87	3.0	1.03	3.8	1.32	3.4	1.10
Total	Non-pattern	4.4	1.39	4.0	1.14	3.3	0.89	3.0	1.04	3.1	1.16	3.1	0.90
	Pattern	4.6	1.21	3.6	0.93	3.3	0.95	3.3	1.07	3.9	1.16	3.7	1.30
	Total	4.5	1.30	3.8	1.05	3.3	0.92	3.1	1.06	3.5	1.21	3.4	1.15

**Table 4:** Simple effects in novelty.

Interaction	Variables	I	J	Mean Difference (I–J)	F	*p*	η^2^
Shape × Decoration	Non-pattern	Non-hat	Hat	−0.460	1.739	0.191	0.022
	Pattern	Non-hat	Hat	0.530	2.308	0.133	0.029
	Non-hat	Non-pattern	Pattern	−1.115	10.217	0.002	0.119
	Hat	Non-pattern	Pattern	−0.125	0.128	0.721	0.002

**Table 5:** Descriptive statistics for time to first fixation.

Variable		M	SD	N
Non-hat	Non-pattern	2.7	0.55	20
	Pattern	2.9	0.69	20
	Total	2.8	0.62	40
Hat	Non-pattern	2.7	0.46	20
	Pattern	1.7	0.47	20
	Total	2.2	0.69	40
Total	Non-pattern	2.7	0.50	40
	Pattern	2.3	0.85	40
	Total	2.5	0.73	80

**Table 6:** Simple effects in time to first fixation.

Interaction	Variables	I	J	Mean Difference (I–J)	F	*p*	η^2^
Shape × Decoration	Non-pattern	Non-hat	Hat	0.050	0.082	0.775	0.001
	Pattern	Non-hat	Hat	1.225	49.473	<0.001	0.394
	Non-hat	Non-pattern	Pattern	−0.140	0.646	0.424	0.008
	Hat	Non-pattern	Pattern	1.035	35.316	<0.001	0.317

**Table 7:** Descriptive statistics for first-pass total fixation duration.

Variable		M	SD	N
Non-hat	Non-pattern	1.1	0.34	20
	Pattern	2.1	0.61	20
	Total	1.6	0.71	40
Hat	Non-pattern	1.8	0.75	20
	Pattern	2.5	0.69	20
	Total	2.1	0.79	40
Total	Non-pattern	1.4	0.67	40
	Pattern	2.3	0.67	40
	Total	1.9	0.79	80

**Table 8:** Descriptive statistics for second-pass total fixation duration.

Variable		M	SD	N
Non-hat	Non-pattern	3.8	1.52	20
	Pattern	5.6	1.32	20
	Total	4.7	1.66	40
Hat	Non-pattern	5.4	1.11	20
	Pattern	7.4	1.72	20
	Total	6.4	1.77	40
Total	Non-pattern	4.6	1.54	40
	Pattern	6.5	1.78	40
	Total	5.5	1.91	80

## Data Availability

The raw data supporting the conclusions of this article will be made available by the authors upon request.
